# Role of preoperative biliary stents, bile contamination and antibiotic prophylaxis in surgical site infections after pancreaticoduodenectomy

**DOI:** 10.1186/s12876-016-0460-1

**Published:** 2016-03-31

**Authors:** Francesca Gavazzi, Cristina Ridolfi, Giovanni Capretti, Maria Rachele Angiolini, Paola Morelli, Erminia Casari, Marco Montorsi, Alessandro Zerbi

**Affiliations:** Pancreatic Surgery Unit, Department of Surgery, Humanitas Research Hospital, Via Manzoni 56, 20089 Rozzano, Milan Italy; Infectious Diseases Unit, Hospital Health Direction, Humanitas Research Hospital, Rozzano, Italy; Microbiology Unit, Analysis Laboratory, Humanitas Research Hospital, Rozzano, Italy; Chancellor of Humanitas University, Chief of Department of Surgery, Humanitas Research Hospital, Rozzano, Italy

**Keywords:** Stent, Pancreaticoduodenectomy, Surgical site infection, *Enterococcus spp*

## Abstract

**Background:**

The routine use of preoperative biliary drainage before pancreaticoduodenectomy (PD) remains controversial. This observational retrospective study compared stented and non-stented patients undergoing PD to assess any differences in post-operative morbidity and mortality.

**Methods:**

A total of 180 consecutive patients who underwent PD and had intra-operative bile cultures performed between January 2010 and February 2013 were retrospectively identified. All patients received peri-operative intravenous antibiotic prophylaxis, primarily cefazolin.

**Results:**

Overall incidence of post-operative surgical complications was 52.3 %, with no difference between stented and non-stented patients (53.4 % vs. 51.1 %; *p = 0.875*). However, stented patients had a significantly higher incidence of deep incisional surgical site infections (SSIs) (*p = 0.038*). In multivariate analysis, biliary stenting was confirmed as a risk factor for deep incisional SSIs (*p = 0.044*). Significant associations were also observed for cardiac disease (*p = 0.010*) and BMI ≥25 kg/m^2^ (*p = 0.045*). *Enterococcus spp.* were the most frequent bacterial isolates in bile (74.5 %) and in drain fluid (69.1 %). In antimicrobial susceptibilty testing, all Enterococci isolates were cefazolin-resistant.

**Conclusion:**

Given the increased risk of deep incisional SSIs, preoperative biliary stenting in patients underging PD should be used only in selected patients. In stented patients, an antibiotic with anti-enterococcal activity should be chosen for PD prophylaxis.

## Background

Obstructive jaundice is the most frequent clinical presentation of a periampullary tumor (i.e., in the the pancreatic head, distal bile duct or the ampulla of Vater). Preoperative biliary drainage has been widely used to provisionally resolve the problem but its routine implementation has become controversial and its diagnostic or therapeutic benefit is no longer clear [1]. It has been argued that stenting patients with jaundice before pancreatic surgery may be beneficial [2], since pathological changes associated with increased bilirubin (e.g., itching, endotoxemia, bleeding problems, hepatic insufficiency) [3] and the retention of bile salts (e.g., a negative chronotropic cardiac effect and increased sodium renal excretion, resulting in hypovolemia, hypotension and decreased renal perfusion) [4] can compromise the outcome of surgery. Moreover, on a practical level, preoperative biliary drainage can be done at less specialized centers without the facility to conduct immediate pancreatic surgery, allowing later scheduling of surgery in a high-volume hospital [5–8]. Preoperative biliary stenting also allows patients to be candidated for neoadjuvant therapies [9].

However, there is evidence that endoscopic and/or percutaneuos biliary drainage increases the risk of bacterial colonization of the bile, since biliary stenting disrupts the mechanical barrier function of the Oddi’s sphincter and predisposes to ascending infection from the duodenum [10, 11]. Bile colonization has been associated with increased incidence of infectious post-operative complications, which may worsen surgical morbidity and mortality [12]. Some authors have reported a significantly greater incidence of post-operative wound infections in stented patients [13, 14], particularly after pancreaticoduodenectomy (PD) [4, 15].

This observational retrospective study compared stented and non-stented patients undergoing PD to assess any differences in post-operative morbidity and mortality. In addition, intra-operative bile and abdominal drain fluid cultures were microbiologically assessed, and the susceptibility of bacterial isolates to antibiotics currently used for preoperative short-term prophylaxis evaluated [16].

## Methods

A prospectively collected database of patients who underwent pancreatic surgery at the Humanitas Research Hospital, Rozzano, Italy, between January 2010 and February 2013 was reviewed and 187 consecutive patients who underwent PD for benign or malignant disease were identified. All patients provided written consent and this retrospective study was approved by the ethics committee of our center (Comitato Etico Indipendente IRCCS Istituto Clinico Humanitas) the 7th of July 2015. Of these patients seven were excluded from the analysis; five because technical failures resulted in intra-operative bile cultures not being available and two because surgery on the biliary tree occurred before PD. Demographic and clinical characteristics of patients were reported, including the occurrence of previous cancers and presence of co-morbidities (cardiac disease or diabetes mellitus).

All patients received peri-operative intravenous (IV) antibiotic prophylaxis in accordance with Italian and international guidelines on antimicrobial prophylaxis in surgery [16, 17]. The majority received IV cefazolin 2 g 30 to 60 min before cutaneous incision with intra-operative doses repeated every 3 h to a maximum of four injections. An intra-operative bile specimen was routinely taken before transection of the biliary tree and sent immediately for microbiological evaluation (aerobic bacteria and fungi). To minimize intra-peritoneal accumulation of bile and reduce contamination of the surgical field, the transected common hepatic duct was temporarily occluded until biliary-enteric continuity was restored by end-to-side anastomosis. Moreover, the peritoneal cavity and wound, both the fascial and subcutaneuos planes, were throughly rinsed at the end of surgery [18]. Superior mesenteric or portal venous resection was done in seven patients (3.9 %). Reconstruction was performed by end-to-side double-layer pancreaticojejunostomy, when possible duct-to-mucosa. End-to-side hepaticojejunostomy was carried out 10–15 cm distal to the pancreatic anastomosis. A pylorus-preservation procedure was usually performed, and an end-to-side antecolic duodenojejunostomy was realized 30 cm distal to the biliary-enteric anastomosis. At the end of the surgical procedure, two 12 mm Penrose type drainages (Redax®, Poggio Rusco, MN, Italy), were placed to the front and rear of the pancreaticojejunostomy with another running under the biliary-enteric anastomosis. Octreotide was routinely administrated during the post-operative course.

From September 2010, a microbiological surveillance culture was taken on the 5th post-operative day (POD5) with fluid from the rear drain. This culture was repeated if there was clinical evidence of intra-abdominal infection i.e., a ‘sinister appearance’ of drained fluid or outflow from a positioned percutaneous pig-tail tube (*n* = 23). Twenty-four (13.3 %) patients lacked a POD5 culture from the rear pancreatic drainage (operated on before September 2010, *n* = 15; rear pancreatic drainage empty, *n* = 7; early death, *n* = 2).

Post-operative morbidity, mortality (death within 30 days after day of hospital discharge), reintervention and readmission rates were evaluated overall and for stented and non-stented groups. Post-operative complications included pancreatic, biliary, duodenal/gastric and lymphatic fistulas, abdominal abscess, delayed gastric empting (DGE), wound infection, post-pancreatectomy hemorrhage (PPH), pancreatitis, urinary tract infection, sepsis and hospital-acquired pneumonia. Post-operative pancreatic fistula (POPF), PPH and DGE were defined according to International Study Group for Pancreatic Surgery recommendations [19–21]. Biliary and lymphatic fistulas were defined by the presence of bile or lymph, respectively, in surgical drains. The presence of fluid collection on computed tomography (CT) was required to confirm intra-abdominal abscess. A diagnosis of infection required fever as well as elevation of white blood cell count and C-reactive protein. Wound infection required purulent drainage from the surgical incision with bedside opening of the wound. Wound infections were further investigated using the definitions of surgical site infections (SSIs) according to the Centers for Disease Control and Prevention (CDC) [22] and Infection Diseases Society of America (IDSA) guidelines [23]. Pancreatitis was diagnosed if patients had abdominal pain, elevated serum amylase level and CT signs. Urinary tract infection was determined by a positive urine culture. Sepsis required ≥2 of fever >38.5 °C or <35 °C, heart rate >90 bpm, respiratory rate >20 breaths per minute, and a confirmed infection. A diagnosis of pneumonia required radiographic evidence of an infiltrate.

### Microbiological cultures

Microbiological cultures of the bile and drainage fluid were performed by conventional methods. In vitro antimicrobial susceptibility of isolated organisms was done in a local microbiology laboratory using the Phoenix 100 automated system (Becton Dickinson, Spark, MD, USA) according to EUCAST interpretation criteria.

### Statistical analysis

Demographic and clinical characteristics were described as frequencies and proportions or as median and range. Differences between stented and non-stented groups were evaluated using the Chi-square test or the Fisher exact test, when appropriate, for categorical data. Mean and 95 % confidence intervals (95 % CIs) were calculated to summarise continuous data and groups were compared by *t*-test. The risk of deep incisional SSIs associated with a range of demographic and clinical characteristics was analysed with univariate and multivariable logistic regression models being used to estimate adjusted odds ratios (OR) and corresponding 95 % CIs. In multivariate analysis we consider only the characteristics statistically significant at the univariate analysis. Statistical significance level was set at 0.1 for univariate and 0.05 for multivariate analysis. All *p*-values were two-sided. Statistical analysis was performed using STATA v.13 software.

## Results

### Patients

Demographic and clinical characteristics of the 180 patients are shown in Table 1.

**Table 1:** Demographic and clinical characteristics

Characteristics	All	Non-stented patients	Stented patients	*P*-value
	(*N* = 180)	(*N* = 91, 50.6 %)	(*N* = 89, 49.4 %)	
Sex: Female/Male	72/108	40 (55.6)/51 (47.2)	32 (44.4)/57 (52.8)	0.346
Age: <68/≥68 (years)	85/95	44 (51.8)/47 (49.5)	41 (48.2)/48 (50.5)	0.875
Body mass index: <25/≥25 (kg/m^2^)	112/68	56 (50.0)/35 (51.5)	56 (50.0)/33 (48.5)	0.970
Previous neoplasia: No/Yes	143/37	74 (51.8)/17 (46.0)	69 (48.2)/20 (22.5)	0.657
Surgery for neoplasia: No/Yes	25/155	24 (96.0)/67 (43.2)	1 (4.0)/88 (56.8)	<0.001
Diabetes: No/Yes	144/36	73 (50.7)/18 (50.0)	71 (49.3)/18 (50.0)	1.000
Preoperative jaundice: No/Yes	120/60	78 (65.0)/13 (21.7)	42 (35.0)/47 (78.3)	<0.001
Cardiac disease: No/Yes	155/25	79 (51.0)/12 (48.0)	76 (49.0)/13 (52.0)	0.952
ASA physical status: 1-2/3-4	124/56	66 (53.2)/25 (44.6)	58 (46.8)/31 (55.4)	0.365
Previous antibiotic therapy: No/Yes	166/14	85 (51.2)/6 (42.9)	81 (48.8)/8 (57.1)	0.748
Preoperative CT or RT: No/Yes	170/10	88 (51.8)/3 (30.0)	82 (48.2)/7 (70.0)	0.210*

ASA *American Society of Anesthesiologists*, CT *chemotherapy*, RT *radiotherapy*, *Fisher exact test

Sixty-percent of patients were male (*n* = 108), median age was 68 years (range 35–84) and median body mass index (BMI) was 23.5 kg/m^2^ (range 15.2–36.1). Thirty-seven patients (20.6 %) had a previous malignant neoplasm, 36 (20.0 %) had diabetes and 25 (13.9 %) had cardiac disease.

A total of 102 patients at diagnosis (56.7 %) had obstructive jaundice with a median total bilirubin of 12.14 mg/dl. Of these, 89 underwent preoperative biliary drainage; 75 (84.4 %) had a plastic stent, six (6.7 %) had a metal stent, two (2.2 %) underwent endoscopic retrograde cholangiopancreatography (ERCP) with nasal-biliary tubes and six (6.7 %) had a percutaneous transhepatic catheter inserted. Median duration of stent placement in the biliary tree was 35 days (range 8–393). Twenty-four stented patients (27 %) needed more than one ERCP. Fifty-nine patients (66.3 %) were referred to our institute with the stent in place; we decided to drain the other 30 jaundiced patients, considering our elective surgery waiting list, for the impossibility to plan their operation in a short period of time. In thirteen cases favourable conditions allow us to operate jaundice patients before stenting. In our series there wasn’t any clinical selection bias on icteric patients to drain.

The other 13 patients with jaundice (median total bilirubin 10.02 mg/dl) received no treatment. In total, 91 patients had no biliary stent; of these, six patients underwent diagnostic ERCP while the other did not undergo any pre-operative manoeuvre on the biliary tract. A total of 60 patients (33 %), of which 47 stented patients, still were icteric (total bilirubin > 2.5 mg/dl) before surgery (Table 1).

The demographic and clinical characteristics of patients with and without pre-operative biliary stents were similar, with the exception that post-operative diagnoses revealed that significantly more of the stented group had a malignant neoplasm (56.8 % vs 43.2 % of non-stented patients; *p < 0.001*). Post-operative diagnoses are summarized in Table 2.

**Table 2:** Post-operative diagnosis of disease

Post-operative diagnosis	N	%
Malignant disease	155	100
Pancreatic ductal adenocarcinoma	75	48.4
Vater papilla adenocarcinoma	30	19.4
Invasive IPMN	17	11.0
Common bile duct adenocarcinoma	16	10.3
NET	13	8.4
Duodenal adenocarcinoma	3	1.9
Pancreatic liposarcoma	1	0.6
Benign disease	25	100
IPMN	13	52.0
Chronic pancreatitis	9	36.0
Duodenal/Vater papilla adenoma	3	12.0

### Surgical characteristics

Mean estimated blood loss during surgery was 406.1 ml (95 % CI: 337.5, 474.7) in all patients; 444.9 ml (95 % CI: 320.5, 569.4) in the stented group and 368.1 ml (95 % CI: 306.1, 430.1) in the non-stented group (*p = 0.274*). Mean operative time was 458.5 min (95 % CI: 448.1, 468.8), with similar mean durations in stented and non-stented patients (454.8 min [95 % CI: 438.8, 470.8] vs. 462.1 min [95 % CI: 448.6, 475.6]; *p = 0.489*). A total of 141 patients (78.5 %) did not receive any blood transfusion, with no difference between patients with and without stents (*p = 0.424*). The mean calculated transfusion volumes were 150.4 ml (95 % CI: 95.2, 205.7) for all patients; 117 ml (95 % CI: 60.7, 174.5) in the stented group and 183.9 ml (95 % CI: 87.7, 280.1) in the non-stented group (*p = 0.274*).

### Post-operative outcomes

Overall, five patients died (2.8 %) during the follow-up period, three in the stented group (3.4 %) (one due to early septic shock and two because of multi-organ failure following a type C fistula) and two in the non-stented group (2.2 %) (one due to hemorrhagic stroke and one due to aspiration pneumonia). In the post-operative period, 14 patients (7.8 %) required reintervention, eight (9.0 %) in the stented group and six (6.7 %) in the non-stented group (*p = 0.764*). Reasons for reintervention were early bleeding due to technical failure (*n* = 4, 28.6 %), pancreatic fistula type C (*n* = 3, 21.4 %), duodenal anastomotic dehiscence (*n* = 3, 21.4 %), biliary anastomotic dehiscence, sepsis, occlusion, late bleeding and fascial dehiscence (all *n* = 1, 7.1 %). Twelve patients (6.7 %) were readmitted to our hospital after discharge: three patients (3.4 %) in the stented group and nine (9.9 %) in the non-stented group (*p = 0.133*).

The overall incidence of post-operative complications was 61.7 % (*n* = 111), with no significant difference between stented and non-stented patients (65.2 % vs. 58.2 %; *p = 0.422*). Incidence of surgical post-operative complications (i.e., pancreatic, biliary, duodenal and lymphatic fistulas, abdominal abscess, DGE, wound infection and PPH) was 52.3 % (*n* = 93) overall, 53.4 % in stented patients and 51.1 % in non-stented patients (*p = 0.875*). Of the 111 patients with complications, 67 (60.4 %) had Clavien scores ≤2 and 69 (38.3 %) had an uneventful post-operative course. Post-operative complications are summmarized in Table 3.

**Table 3:** Postoperative complications stratified for the presence of biliary stent (Univariate analysis)

Complications	All (*N* = 178)	Non-stented (*N* = 90)	Stented (*N* = 88)	*P* value
	N (% col)	N (% col)	N (% col)	
Pancreatic fistula: No/Yes	127 (71.3)/51 (28.7)	64 (71.1)/26 (28.9)	63 (71.6)/25 (28.4)	1.000
A	19 (10.7)	10 (11.1)	9 (10.2)	0.995^a^
B + C	32 (18.0)	16 (17.8)	16 (18.2)	
Biliary fistula: No/Yes	167 (93.8)/11 (6.2)	82 (91.1)/8 (8.9)	85 (96.6)/3 (3.4)	0.212^b^
Abdominal abscess: No/Yes	170 (95.5)/8 (4.5)	85 (94.4)/5 (5.6)	85 (96.6)/3 (3.4)	0.720^b^
Pneumonia: No/Yes	170 (95.5)/8 (4.5)	85 (94.4)/5 (5.6)	85 (96.6)/3 (3.4)	0.720^b^
Urinary tract infection: No/Yes	175 (98.3)/3 (1.7)	88 (97.8)/2 (2.2)	87 (98.9)/1 (1.1)	1.000^b^
Wound infection: No/Yes	141 (79.2)/37 (20.8)	78 (86.7)/12 (13.3)	63 (71.6)/25 (28.4)	0.022
Pancreatitis: No/Yes	175 (98.3)/3 (1.7)	87 (96.7)/3 (3.3)	88 (100)/0 (0.0)	0.246^b^
Sepsis: No/Yes	172 (96.1)/7 (3.9)	87 (96.7)/3 (3.3)	85 (95.5)/4 (4.5)	0.720^b^
Lymphatic fistula: No/Yes	168 (94.4)/10 (5.6)	87 (96.7)/3 (3.3)	81 (92.0)/7 (8.0)	0.209^b^
Post-pancreatectomy hemorrhage: No/Yes	153 (86.0)/25 (14.0)	76 (84.4)/14 (15.6)	77 (87.5)/11 (12.5)	0.711
A	14 (7.9)	9 (10.0)	5 (5.7)	0.958^a^
B	1 (0.5)	1 (1.1)	0 (0.0)	
C	10 (5.6)	4 (4.4)	6 (6.8)	
Duodenal/gastric fistula: No/Yes	175 (98.3)/3 (1.7)	89 (98.9)/1 (1.1)	86 (97.7)/2 (2.3)	0.619^b^
Delayed gastric emptying: No/Yes	173 (97.2)/5 (2.8)	88(97.8)/2 (2.2)	85 (96.6)/3 (3.4)	0.680^b^

^a^Considering the subgroups

^b^Fisher exact test

Two patients were excluded from analysis of post-operative complications because of early death (respectively 3rd and 5th post-operative day)

Types of post-operative complications were generally similar between stented and non-stented patients. Pancreatic fistula, including clinically significant type B and C fistula, occurred at a similar rate in stented and non-stented patients. Wound infection was the only complication which showed a statistically significant difference between groups, being reported in 28.4 % of stented patients compared with 13.3 % of non-stented patients (*p < 0.022*). Stented patients had a significantly higher incidence of deep incisional SSIs (13.6 % vs 4.4 %; *p = 0.038*), whereas the incidence of superficial incisional SSIs and organ/space SSIs was not significantly different between the two groups (14.8 % vs 8.9 %; *p = 0.325* and 31.5 % vs 22.2 %; *p = 0.163,* respectively) (Table 4).

**Table 4:** Surgical site infections (SSIs) stratified for the presence of stent

Complications	All	Non-stented patients	Stented patients	P value
	N (% col)	N (% col)	N (% col)	
SSI superficial: No/Yes	157 (88.2)/21 (11.8)	82 (91.1)/8 (8.9)	75 (85.2)/13 (14.8)	0.325
SSI deep: No/Yes	162 (91.0)/16 (9.0)	86 (95.6)/4 (4.4)	76 (86.4)/12 (13.6)	**0.038**
SSI organ/space: No/Yes	131 (73.2)/48 (26.8)	70 (77.8)/20 (22.2)	61 (68.5)/28 (31.5)	0.163

Bold data are statistical significant results

Neither the presence of cholangitis and multiple preoperative ERCP, nor high duration of the stent placement in the biliary tree affect the incidence of deep incisional SSIs (*p = 1.000*, *p = 1.000* and *p = 0.532* respectively).

In jaundiced patiets we didn’t find any statistical difference for the incidence of deep SSIs between stented (*n* = 88) and non stented (*n* = 13) (13.6 % vs 7.7 %; *p = 1.000*). We also didn’t find any difference for deep incisional SSIs in patients underwent ERCP without stenting (*n* = 6) compared to non stented patients (*n* = 84) (16.7 % vs 3.6 %; *p = 0.245*). This results could be influenced by the small size of the two subgroups analyzed.

The mean duration of preoperative time was 54.6 days (95 % CI: 42.1,67.1) in the stented group and 32.0 days in the non-stented group (95 % CI: 22.8,41.2), with statistical significant difference between the two groups (*p = 0.004*). However the waiting time for the surgical procedure wasn’t statistically associated with the occurrence of deep incisional SSIs (OR:1.00 95 % CI:1.00,1.01; *p = 0.585*).

In an univariate analysis, cardiac disease (*p = 0.006*), ASA physical status 3–4 (*p = 0.027*) and BMI ≥25 kg/m^2^ (*p = 0.043*) were significantly associated with the occurrence of deep incisional SSIs. No other associations between patient characteristics and deep incisional SSIs were observed, neither for patients affected by neoplasia nor for jaundiced patients before surgery (Table 5). Multivariate analysis confirmed that the presence of a stent in the biliary tree significantly increased the risk of deep incisional SSIs, with stented patients at 3.47 times higher risk than non-stented patients (95 % CI: 1.03, 11.67, *p = 0.044*). Cardiac disease (OR 4.75, 95 % CI: 1.45, 15.53, *p = 0.010)* and BMI ≥25 kg/m^2^ (OR 3.11, 95 % CI: 1.03, 9.42, *p = 0.045*) were also independently significantly associated with an increased risk of deep incisional SSIs in the multivariate model (Table 6).

**Table 5:** Deep SSIs distribution and univariate odds ratio stratified for principal clinical and demographic characteristics

Characteristics	Deep SSIs	Deep SSIs	OR (95 % Confidence Interval)	*P* value
	No	Yes		
	N (% row)	N (% row)		
Sex				
Female Male	63 (87.5) 99 (93.4)	9 (12.5) 7 (6.6)	2.02 (0.72;5.70)	0.184
Age (years)^a^				
<68 ≥68	78 (91.8) 84 (90.3)	7 (8.2) 9 (9.7)	1.19 (0.42,3.36)	0.737
Body mass index				
<25Kg/m^2^ ≥25Kg/m^2^	104 (94.6) 58 (85.3)	6 (5.4) 10 (14.7)	2.99 (1.03;8.64)	**0.043**
Previous neoplasia				
No Yes	130 (91.6) 32 (88.9)	12 (8.4) 4 (11.1)	1.35 (0.41;4.48)	0.619
Surgery for neoplasia				
No Yes	23 (92.0) 139 (90.9)	2 (8.0) 14 (9.1)	1.16 (0.25;5.44)	0.852
Diabetes				
No Yes	130 (90.9) 32 (91.4)	13 (9.1) 3 (8.6)	0.94 (0.25;3.49)	0.923
Preoperative jaundice				
No Yes	108 (90.8) 54 (91.5)	11 (9.2) 5 (8.5)	0.91 (0.30;2.75)	0.866
Cardiac Disease				
No Yes	144 (93.5) 18 (75.0)	10 (6.5) 6 (25.0)	4.80 (1.56;14.78)	**0.006**
ASA physical status				
1-2 3-4	116 (94.3) 46 (83.6)	7 (5.7) 9 (16.4)	3.24 (1.14;9.22)	**0.027**
Previous antibiotic therapy				
No Yes	149 (90.9) 13 (92.9)	15 (9.1) 1 (7.1)	0.76 (0.09;6.25)	0.802
Preoperative Chemotherapy/RT				
No Yes	152 (90.5) 10 (100)	16 (9.5) 0	/	0.306
Preoperative time^a^				
≤28.5 days >28.5 days	85 (94.4) 77 (87.5)	5 (5.6) 11 (12.5)	2.43 (0.81;7.30)	0.114

^a^Age and Preoperative time were cathegorized considering the median value; /:not evaluable

Bold data are statistical significant results

**Table 6:** Odds ratio with their corresponding 95 % confidence intervals for Multivariate final analyses

Characteristics	OR	CI 95 %	*P* value
Stent _Yes vs No_	3.47	1.03;11.67	0.044
Cardiac disease _Yes vs No_	4.75	1.45;15.53	0.010
BMI _≥25 vs <25 Kg/m_^2^	3.11	1.03;9.42	0.045

Odds ratio with their corresponding 95 % confidence intervals for statistically significant factors in the final multivariable model with variables confirming an independent effect

The occurrence of deep incisional SSIs significantly prolonged mean post-operative length of hospital stay to 20.8 days (95 % CI: 14.9, 26.6) compared with 14.0 days (95 % CI: 12.7, 15.3) in patients without deep incisional infections (*p = 0.003*).

### Microbiological assessment

A total of 154 patients (85.5 %) received prophylactic IV cefazolin 2 g, seven (27 %) patients allergic to β-lactams received IV clindamycin 600 mg and 19 (73 %) patients received other antibiotic regimens (cephalosporins, penicillins and/or fluoroquinolones), usually started at home or at a different hospital.

Overall, 106 (58.9 %) intra-operative bile cultures were positive, of which 81 were polymicrobial. Ninety-seven patients (53.9 %) had a positive culture of surgical or percutaneous positioned drainage fluid. Fifteen of 31 patients (48.4 %) with positive intra-operative bile culture and pancreatic fistula revealed the same microrganisms in both in biliary culture and in drainage fluid from the rear of the pancreatic anastomosis on POD5. Microorganisms isolated in bile and drain samples are reported in Table 7. *Enterococcus spp.* (74.5 %) and the Gram-negative *Escherichia coli* (36.8 %), *Klebsiella spp.* (34.9 %), *Enterobacter spp.* (17.9 %) and *Citrobacter spp.* (9.4 %) were the most frequently cultured bacteria in bile specimens; these species were also present in drain fluid cultures, although, with the exception of *Enterobacter spp.,* less frequently than in bile. Gram-positive *Staphylococci spp.* were more frequently isolated from drain fluid than bile (26.8 % vs 4.7 %).

**Table 7:** Microbiological isolates in bile and drain fluid cultures of all patients

Microorganism	Bile				Drain^a^			
	Patients	CEF res^b^	AMP res^c^	*P* value^d^	Patients	CEF res^b^	AMP res^c^	*P* value^d^
	N (%)	%	%		N (%)	(%)	(%)	
*Enterococcus spp.*	79 (74.5)	100	13.9	<0.001	67 (69.1)	100	28.9	<0.001
*Escherichia coli*	39 (36.8)	59	41	<0.113	26 (26.8)	76.9	57.7	<0.139
*Klebsiella spp.*	37 (34.9)	35.1	21.6	0.197	18 (18.6)	72.2	50	0.171
*Enterobacter spp.*	19 (17.9)	94.7	94.7	1.000	24 (24.7)	95.8	100	0.312
*Citrobacter spp.*	10 (9.4)	80	80	-	6 (6.2)	100	83.3	-
*Hafnia alvei*	7 (6.6)	71.4	100	-	3 (3.1)	100	100	-
*Staphylococcus spp.*	5 (4.7)	40	40	-	26 (26.8)	73.1	92.3	0.067
*Pseudomonas aeruginosa*	4 (3.8)	100	100	-	8 (8.3)	100	100	-
*Aeromonas spp.*	3 (2.8)	66.7	33.3	-	0	/	/	-
*Serratia Marcescens*	2 (1.9)	100	100	-	4 (4.1)	100	100	-
*Streptococcus spp.*	2 (1.9)	100	50	-	3 (3.1)	66.7	0	-
*Morganella morganii*	1 (0.9)	100	100	-	2 (2.1)	100	100	-
*Proteus spp.*	1 (0.9)	100	100	-	2 (2.1)	100	50	-
*Candida spp.*	9 (8.5)	/	/	/	21 (21.7)	/	/	/

^a^Drains with positive culture (surgical and percutaneous)

^b^Cefazolin resistance

^c^Ampicillin-sulbactam resistance

^d^Correlation between cefazolin resistance and Ampicillin-sulbactam resistance

- Data were not compared due to the small sample size;/Not evaluable

*Candida spp.* were isolated in 8.5 % of bile cultures, but none of the 17 non-stented patients with positive intra-operative biliary cultures had *Candida* colonization (*p = 0.349*). *Candida spp.* were also more frequently cultured from drain fluid than from bile (21.7 % vs 8.5 %).

High levels of resistance to cefazolin were observed, with resistance to ampicillin-sulbactam also reported (Table 7). Extended-spectrum beta-lactamase (ESBL)-producing Gram-negative bacteria, including *E. Coli*, *Enterobacter* spp., *Citrobacter* spp., *Klebsiella* spp. and *Serratia marcescens* were isolated in bile and drain cultures: 80 patients had these bacteria present in bile cultures, 17.5 % (*n* = 14) of which were ESBL-producing strains.; 62 patients had these bacteria cultured in their drain effluent, 32.2 % (*n* = 20) of which were ESBL-producing strains.

All stented patients had positive bile cultures (*n* = 89, 100 %) compared with only 18.7 % (*n* = 17) of non-stented patients (*p* < 0.001). *Enterococcus spp*. were significantly more frequent in bile samples from stented patients compared with non-stented patients with positive intra-operative bile specimens (78.9 % vs 52.9 %; *n = 0.026*). ESBL-positive strains were isolated only in intra-operative bile cultures of stented patients.

We observed that all the PTBD patients (100 %) had infected intraoperative bile and 4 (66.6 %) out of 6 patients with PTBD had their bile culture polymicrobic (3 germs each).

## Discussion

The aim of this study was to assess the risk of post-operative complications associated with preoperative biliary stenting in patients undergoing pancreatic head resection. Consistent with several previous studies, no differences in mortality or overall morbidity were observed between stented and non-stented patients [2, 9, 15, 24]. The mortality rate of 2.8 % is similar to that reported by other high-volume pancreatic surgery centers [19]. Overall morbidity was high (61.7 %), but only 39.6 % of patients had serious complications (Clavien-Dindo classification grade ≥3). Surgical morbidity was 52.3 %, which is also consistent with that previously described [25–27].

The incidence of the most common post-operative complications, including pancreatic fistula, biliary fistula and abscess, were not significantly different between stented and unstented groups. However, there was a statistically significant higher incidence of wound infection in patients with stents compared with non-stented patients, a finding that has also been reported in other studies. In a retrospective review of 567 patients undergoing PD without prior operative biliary bypass, stented patients had a significantly higher incidence of wound infection compared to patients without stents (10 % vs. 4 %; *p = 0.02*) [15]. A study of 212 patients presenting with obstructive jaundice who subsequently underwent PD reported that wound infection occurred in 8 % of patients with preoperative drainage but was not observed in any non-stented patients (*p = 0.039*) [4]. Similarly, Howard and colleagues reported that preoperative biliary stenting increased the incidence of wound infection in a study of 138 patients with obstructive jaundice (28.4 % vs 13.3 %; *p < 0.022*) [28], while Limongelli et al. also found wound infections significantly more frequent in stented compred with unstented patients [13]. In another study of 300 consecutive PD patients, there was a significant increase in wound infections in patients treated by preoperative prosthetic biliary decompression or surgical biliary bypass (*n* = 13) compared with non-stented patients (*n* = 4; *p = 0.022*) [14]. However, in a study conducted at the same centre 4 years later, the presence of an endobiliary stent of any kind (metal or plastic) was not associated with increased

PD-associated mortality or morbidity, including wound infection [9]. The authors suggested that the difference in superficial wound infection rates between the two studies may have been related to differences in attentiveness to wound irrigation and closure.

A trend to an higher rate of postoperative infection is also observed, in our population, in patient that underwent pancreaticoduodenectomy for neoplasia. This could be explained by the higher rate of stented patients among the ones that have a pancreatic neoplasia (56 % vs 4 % *p* < 0.001).

To better understand the observed difference in wound infection rate between stented and non-stented patients in this study, we further analysed this complication using the CDC National Nosocomial Infection Surveillance System (NNIS) standardized surveillance criteria to define wound infections as superficial incisional SSIs, involving only the skin and subcutaneous tissue of the incision, or deep incisional SSIs involving deep soft tissues of the incision (e.g., fascial and muscle layers) [22]. No differences were observed beween stented and non-stented groups in the incidence of superficial wound infections, but stented patients had a 3.47-fold higher risk of developing deep incisional infections. The presence of a deep incisional SSI was associated with a significant increase in hospital length of stay, which increased from two to almost three weeks. The negative impact of SSIs on hospital length of stay and healthcare costs following complex hepatopancreatobiliary operations has previously been reported [18]. Moreover, deep incisional SSIs increase the risk of immediate or late fascial dehiscence. During follow-up of the 16 patients with deep incisional SSIs in this study, six (37.5 %) developed an incisional hernia.

In multivariate analysis, cardiac disease and a BMI ≥25 kg/m^2^ were independent risk factors for the occurrence of deep incisional SSIs. It has long been known that ‘past and present heart trouble’ significantly increases the morbidity of patients undergoing PD, with Gildorf and Spanos reporting in 1973 that 13 (54 %) of 24 patients with cardiac disease had major complication or death after PD. [29] More recently, Lin at al. found that a history of coronary artery disease correlated with increased fistula rate in PD. [30] In the present study, patients with cardiac disease had a 4.75 times higher risk of developing post-operative deep incisional SSIs than patients without cardiac problems. Opinions on the role of obesity as a universal predictor of poor outcomes in surgical patients vary [25]. In many type of surgery, including orthopedic, gynecological, cardiac and colorectal, obesity is an established risk factor for post-operative morbidity [31, 32]. In pancreatic surgery, Su at al [33]. reported that BMI ≥25 kg/m^2^ was an independent predictor of serious post-operative infectious complications, although not wound infection, after PD. However, a multivariate analysis of 356 patients undergoing PD showed that patients with BMI ≥30 kg/m^2^ and especially those with BMI ≥35 kg/m^2^ were at significantly higher risk of post-operative wound infection but not any other complication [31]. While both cardiac disease and BMI were independently associated with an increased risk of developing a deep SSI, they did not negate the independent impact of stenting on the increased incidence of deep SSIs.

All 89 patients with the biliary stent in place before surgery had bacterial contamination of bile compared with only 18.7 % of non-stented patients (*p < 0.001*). Other authors have also reported bactibilia in stented patients but at lower rates than we observed. Howard et al. observed bactibilia in 80 % of stented patients versus 42 % of non-stented patients (*p < 0.0001*) [28], while Jagannath and colleagues found 47 % of patients with pre-operative biliary drainage had a positive bile culture compared with 31 % of those without stents [34]. One possible reason for the high percentage reported in this study could be the period of time between positioning of the biliary stent and surgery. The longer the biliary stent is in place, the longer the reflux of intestinal bacteria into the biliary tree and thus the risk of bacterial colonization. In our cohort, 65 % of stented patients had a stent in place for over 4 weeks and 38.2 % for over 6 weeks, since at least 4–6 weeks is necessary for full recovery of the metabolic abnormalities associated with obstructive jaundice [35]. In addition, 24 (27 %) of the 89 stented patients in this study needed more than one ERCP in order to change a plugged stent that was causing cholangitis while awaiting surgery. The need for more than one endoscopic procedure for jaundice palliation may also increase the risk of bactibilia.

Seventeen non-stented patients also had bacteria present in bile. Bile culture is usually negative in healthy persons [36] and patients with malignant biliary obstruction before biliary decompression, although other studies published before and after the introduction of ERCP have shown bactibilia in the absence of sphincterotomy and biliary drainage and in patients with malignant obstructive jaundice [2, 34, 37]. Among our 17 non-stented patients with bactibilia, only six had diagnostic ERCP before surgery and only one was jaundiced so it is difficult to explain how the positive bile cultures occurred in the 11 non-stented patients. However, this rate is lower than observed in other studies and the presence of bactibilia in 18.7 % of patients without a clear explanation suggests intra-operative bile cultures are justified in all open biliary tract procedures.

The other aim of this study was to evaluate the microbiology of bile and abdominal drainage fluid cultures and to investigate how the two may be related. The microorganisms most commonly isolated in bile cultures were *Enterococcus spp.* (74.5 %) and the Gram-negative bacteria *E. coli* (36.8 %), *Klebsiella spp.* (34.9 %) and *Enterobacter spp* (17.9 %). Dominant growth of *Enterococci* and *Enterobacteriaceae* was observed in both bile and drain fluid cultures. These findings are consistent with other studies that isolated similar bacteria in bile [10, 11, 27, 36, 38]. Polymicrobial colonization of bile was frequent, similar to many previous observations [13, 28, 34, 39, 40]. However, unlike our study, previous investigations have not always isolated *Candida spp*. in bile cultures, although the presence of *Candida spp*. in bile was previously observed by both Howard et al. (21 % of patients) [28] and Karsten et al. (10 %) [41]. While Howard and colleagues reported fungal infection in both stented and non-stented patients, *Candida spp*. were only isolated in bile samples from stented patients (10.1 %) in our analysis. The importance of *Candida spp.* in bile culture should not be overlooked, especially given that, unlike anti-bacterial prophylaxis, anti-fungal prophylaxis is often not administered [42]. Moreover, an increase of *Candida spp*., as well as *Staphylococci spp*., was also observed in cultures from drainage fluid cultures compared with bile. Recently Kent at al [26]. isolated *Candida spp*. (10 %) as well as *Staphylococcus spp*. (18 %) and other

Gram-positive and negative pathogens from infectious complications of pancreatic resection. Hovewer, the higher isolation of *Candida spp.* and *Staphylococci spp.* in drain fluids could be due to the use of Penrose drainage and a possible contamination at the collection site. A surveillance culture from fluid taken from the surgical drain to the rear of the pancreatic anastomosis on POD5 was done in order for early identification of pathogens in case of fistula, as well as to analyze if they were correlated with bacteria isolated from intra-operative bile culture [23]. Lygidakis et al. [43] demonstrated that microrganisms isolated from bile in operated patients could be found in the blood, infected wound exudate and intra-abdominal abscesses of those patients with post-operative complications. Similarly, in a study of 161 patients undergoing PD, Povoski et al. [44] found that microrganisms cultured from bile were predictive of those cultured from subsequent intra-abdominal abscesses (100 % of patients), and wound infection (69 % of patients). Howard et al. [28] found that 42 % of infectious complications cultured the same bacteria as bile. In this study, 48.4 % of patients with positive intra-operative bile cultures and pancreatic fistula had the same pathogens in bile and drain fluid at POD5, a remarkably high rate considering that 74 patients (41.1 %) had negative intra-operative bile cultures and 16 had no evaluable POD5 drain fluid culture.

We also investigated the susceptibility of bacterial isolates to cefazolin, usually administered peri-operatively, and to ampicillin-sulbactam, another antibiotic often recommended for prophylaxis in biliopancreatic surgery [16, 17, 38]. Overall, 154 patients (85.5 %) received preoperative cefazolin. However, high levels of bacterial resistance to this prophylactic regimen were observed. All *Enterococci* isolates were resistant to cefazolin and susceptibilty among other bacteria varied from zero to 65 %. *Enterococcus spp*. were the bacteria most frequently cultured in bile (74.5 % of patients) and in drain effluent (69.1 %) and anti-enterococcal activity is essential for effective peri-operative antibiotic therapy. As such, the choice of prophylactic cefazolin does not apppear to have been appropriate in this cohort. Of note, a higher incidence of *Enterococcus spp*. isolated from preoperative bile cultures approached statistical significance in the stented versus unstented group (78.9 % vs 52.9 %; *p = 0.026*). Resistance to ampicillin-sulbactam was lower, with 86 % of *Enterococcus spp*. isolates from bile samples and 71 % from drain fluid effluent susceptible. Given this, ampicillin-sulbactam might be the preferred antibiotic of first choice in PD prophylactic regimens, rather than being considered as an alternative to cefazolin. Moreover, considering the presence of 17.5 % ESBL Gram-negative bacteria in the bile of stented patients, peri-operative IV prophylaxis should perhaps involve a β-lactamase antibiotic active against *Enterococcus spp*. combined with an antibiotic active against ESBL Gram-negative pathogens.

## Conclusions

Given the increased risk of deep incisional SSIs and the associated delay in discharging patients, preoperative biliary stenting in patients undergoing pancreatic surgery should be selectively rather than routinely used. Jaundiced patients candidate to surgery procedure had to be drained if sepsis prevents operation. In icteric patients unfit for surgery, addressed to neoadjuvant chemotherapy, is necessary to positioning a biliary stent. Local hospital factors, like elective surgical waiting list time, presence of operative endoscopic facility, can influence the decision-making of biliary drainage. It’s desirable to develop guidelines to standardized treatment and care. However, our results suggest that reducing the time patients wait for PD shoud be a higher priority. Patients with cardiac disease or a high BMI are at risk of deep incisional SSIs and require careful peri- and post-operative monitoring. Adjunctive therapies aimed at reducing the risk of deep incisional SSI should be considered in these patients. Pancreaticoduodenectomy requires antibiotic prophylaxis to prevent post-operative infections of the abdominal wall and intra-abdominal tissues: in patients with biliary stents placed for obstructive jaundice and preoperative infected bile an antimicrobial therapy with anti-enterococcal activity should be chosen. Prospective multicentre randomized studies are required to investigate novel antibiotic prophylaxis regimens for PD.

## Abbreviation

BMI: Body mass index
CDC: Center for disease control and prevention
CIs: Confidence intervals
CT: Computed tomography
DGE: Delayed gastric empting
ERCP: Endoscopic Retrograde Colangiopancreatography
ESBL: Extended spectrum beta lactamase
EUCAST: European Committee on Antimicrobial Susceptibility Testing
IDSA: Infection Diseases Society of America
IV: intravenous
NNIS: National Nosocomial Infection Surveillance System
OR: Odds ratios
PD: Pancreaticoduodenectomy
POD: Post-operative day
POPF: Post-operative pancreatic fistula
PPH: PostPancreatectomy hemorrhage
Spp: Species
SSI: Surgical site infections
